# Recognition of visual symptoms in stroke: a challenge to patients, bystanders, and Emergency Medical Services

**DOI:** 10.1186/s12873-023-00870-2

**Published:** 2023-08-25

**Authors:** Kristina Parsberg Berg, Viktor Frederik Idin Sørensen, Stig Nikolaj Fasmer Blomberg, Helle Collatz Christensen, Christina Kruuse

**Affiliations:** 1 Department of Neurology, Copenhagen University Hospital - Herlev Gentofte, Copenhagen, Denmark; 2 Emergency Medical Services Copenhagen, University of Copenhagen, Telegrafvej 5, 2750 Copenhagen, Denmark; 3 Danish Clinical Quality Program (RKKP), National Clinical Registries, Copenhagen, Denmark; 4 Emergency Medical Services Region Zealand, Naestved, Denmark

**Keywords:** Stroke, Visual symptoms, Prehospital, Emergency Medical Services; stroke recognition

## Abstract

**Background:**

Identification of visual symptoms as a sign of acute stroke can be challenging for both first line healthcare professionals and lay persons. Failed recognition of visual symptoms by medical dispatchers at the Emergency Medical Dispatch Center (EMDC-112) or personnel at the Out-of-Hours Health Service (OOHS) may delay stroke revascularization. We aimed to identify correct system response to visual symptoms in emergency calls.

**Methods:**

Phone calls from patient or bystander to the EMDC-112 or OOHS, which included visual symptoms on patients later verified with stroke/Transient ischemic attack (TIA) diagnosis, were analyzed. Data were stratified according to hospitalization within and after 4.5 h from symptom onset. Descriptive and multiple logistic regression analysis were performed.

**Results:**

Of 517 calls identified, 290 calls fulfilled inclusion criteria. Only 30% of the patients received correct visitation by the medical dispatchers and referral to the hospital by a high-priority ambulance. Correct visitation was associated with early contact (adjusted OR: 2.37, 95% CI: 1.11, 5.03), contact to the EMDC-112 (adjusted OR: 3.18, 95% CI: 1.80, 5.62), and when the medical dispatcher asked additional questions on typical stroke symptoms (adjusted OR: 6.36, 95% CI: 3.01, 13.43). No specific visual symptom was associated with stroke recognition and fast hospitalization.

**Conclusions:**

First line healthcare professionals had significant problems in identifying visual symptoms as a sign of acute stroke and eliciting correct response. This highlights an urgent need to improve knowledge of visual symptoms in acute stroke and emphasize correct response to stroke symptoms in general.

**Supplementary Information:**

The online version contains supplementary material available at 10.1186/s12873-023-00870-2.

## Introduction

Stroke is a medical emergency where early assessment and recognition of symptoms are essential for correct acute response and optimal treatment to reduce risk of permanent neurological deficit [1, 2]. Identification of stroke symptoms, especially atypical symptoms, is challenging for each of the patients, bystanders, and first line healthcare professionals. Atypical stroke symptoms include acute visual symptoms, confusion, vertigo, dizziness, and reduced levels of consciousness and these symptoms may often elude early recognition and treatment [1, 3–6]. The typical stroke symptoms are more frequently recognized and include unilateral weakness/hemiparalysis, facial palsy, speech difficulty, and numbness [1, 3–5]. Patients and bystanders are, according to national stroke guidelines, encouraged to call the emergency number 1–1-2 immediately upon recognition of stroke-like symptoms. The call is then directed to the Emergency Medical Dispatch Center (EMDC-112) [7–9]. Notwithstanding this recommendation, stroke patients or bystanders frequently choose the “1813—Medical Helpline”, an Out-of-Hours Health Service (OOHS) established for non-acute cases during out of office hours of the usual general practitioner, as point of first contact [9]. Hence, it is essential that medical dispatchers at both the EMDC-112 and the OOHS can identify acute stroke symptoms, assign the patient a stroke relevant criterion within the reporting system, and initiate immediate transfer to stroke centers with a high-priority ambulance [5, 7, 8]. Occurrence of typical stroke symptoms are associated with increased use of EMDC-112, whereas onset of atypical symptoms less frequently instigates calls to EMDC-112 [3].

There are reports on stroke recognition and handling by the Emergency Medical Services (EMS) in general, but no one have investigated recognition of stroke based on visual symptoms as primary complaint [10–14]. We aimed to investigate how well visual symptoms were recognized as a sign of acute stroke in emergency calls to the EMDC-112 and the OOHS. Further, we aimed to detect how often medical dispatchers elicited a correct chain-of-response to the visual stroke symptoms. We hypothesized, that strokes presenting with mainly visual symptoms were difficult to identify and that this resulted in an incorrect response not complying to current guidelines. We included information on the reported visual symptoms, the prehospital chain-of-response, and factors associated to fast assessment and hospitalization of the stroke incidents.

## Methods

### Study design

In a retrospective cohort study on patients diagnosed with stroke, we included all recorded phone calls to the EMDC-112 or OOHS in the Capital Region of Denmark, in the period January 1^st^, 2016 to December 31^st^, 2018. All included calls concerned patients with a diagnosis of stroke or transient ischemic attack (TIA) verified upon subsequent admission to stroke units registered in DANSTROKE, and with visual symptoms registered in the EMDC-112 or OOHS system as the main complaint. Inclusion criteria were patients above the age of 18 years. Types of visual stroke symptoms were retrieved by listening to all calls and using a structured data form (Telephone Stroke Data Form), created using Research Electronic Data Capture (REDCap) [15, 16]. The visual symptoms were classified into five categories previously applied [17–21]: visual field loss (i.e. complete/partial homonymous hemi anopia, quadrant anopia, scotomas), eye movement abnormalities (i.e. double vision, misaligned eyes, difficulty looking at near objects), reduced central vision (i.e. blurred or altered vision), visual perceptual deficits (i.e. visual neglect, difficulty recognizing faces/objects or processing visual inputs, hallucinations, visual flickering), and total vision loss. Additional data retrieved from the calls included duration, description of the visual symptoms and supplementary symptoms. Further, it was noted if the caller suspected the symptoms to represent stroke, or if the patient previously had a stroke. Finally, we evaluated the response and actions of the medical dispatchers. If the same patient had more than one call to each of the services in the study period, the calls were registered as one if done within 24 h of each other, and two if more than 24-h between calls. Supplementary data were collected from Danish Stroke Registry (DANSTROKE) and the Out-of-hour (OOH) Health Service database. For inclusion in DANSTROKE, the stroke diagnosis and TIA were identified according to the International Statistical Classification of Diseases and Health Related Problems 10^th^ Revision (ICD-10). The stroke diagnosis included both hemorrhagic and ischemic stroke. Symptom onset were collected from DANSTROKE. Onset were recorded in the registry by attending neurologist after discussion with the patient or patients’ relatives or though bystander information on admission, and the estimated time of onset or last seen well were recorded. There were no registration of wake-up stroke. From the population of callers with a verified stroke, callers with registration of visual symptoms, or a mention of visual symptoms in the dispatch notes were identified and labelled as ‘Calls with registration of visual symptoms as main complaints’. A simple free-text search in dispatch notes for words related to vision were used to identify these callers. The free-text search resulted in several records, where ‘eye’ was used in other word-context than describing eye-related symptoms. These were excluded upon listening to the recorded calls.

### Settings

The recorded calls were obtained from the Copenhagen EMS, Denmark, covering a population of approximately 1.8 million inhabitants [9, 22]. The EMDC-112 and OOHS are part of Copenhagen EMS, a coordinated and integrated prehospital emergency healthcare system [22]. In Denmark healthcare including the EMS is tax-financed, free of charge, and equally accessible for all [9, 23]. Citizens are prompted to call the 1–1-2 in life-threatening situations where trained nurses and paramedics are the first responders to the calls. A national criteria-based dispatch protocol, The Danish Index, is used to assess the urgency and severity of the medical situation and to decide the appropriate response. This serves as guideline for correct visitation, which in case of acute stroke is referral to the hospital by a high-priority ambulance [9, 23]. For patients who have shown symptoms of stroke within the last 24 h but are without symptoms at time of call, the guideline for correct visitation is an ambulance within 25 min, but without lights and sirens. In medical situations occurring out-of-office hours of family practitioner, the OOHS may be contacted [9, 22]. The OOHS is operated by physicians and nurses who use a computer-assisted support tool to help determine the appropriate response to the call; telephone advice, referral to consultation at a hospital, a home visit by a physician, or a direct referral to the hospital by an ambulance. The two services differ in call waiting time and use of telephone triage, which has an impact on the stroke chain-of-response and outcome [9, 22, 23].

### Exposure and outcome measures

We had two primary exposures in this study: contact to either the EMDC-112 or OOHS and stroke/TIA diagnosis with registration of visual symptoms as main complaint. Primary outcome was to identify how well visual symptoms were recognized as a sign of acute stroke in emergency calls to the EMDC-112 or OOHS. Secondary outcomes included how many patients were correctly referred to the hospital, and which factors associated to correct stroke response and subsequent referral to the hospital by a high-priority ambulance. We sought to discover which visual symptoms had a stronger association with stroke recognition and fast intervention. To reduce risk of confounders, (age, gender, previous stroke, stroke suspicion etc. retrieved from DANSTROKE), analysis was done with confounders included.

### Statistical analyses

Baseline characteristics and visual symptoms were stratified according to two groups: patients who arrived at the hospital within or after the 4.5-h time-window from stroke onset to revascularization treatment by thrombolysis. Descriptive analyses were performed. Data are given as absolute numbers and percentages, and age is summarized as mean with standard deviation (SD). Missing data shown in tables. Differences in baseline characteristics and visual symptoms were evaluated using Chi-square test and unpaired t-test. Odds ratios (OR) including 95% confidence intervals (CI) were calculated. The association between patients contacting the EMDC-112 or OOHS within 4.5 h from symptom onset and correct visitation by the patients were examined using a multivariate logistic regression model. All statistical analyses were performed with R-Studio and SAS (Statistical Analysis Software, version 9.4, Cary, NC).

## Results

**Fig. 1:**
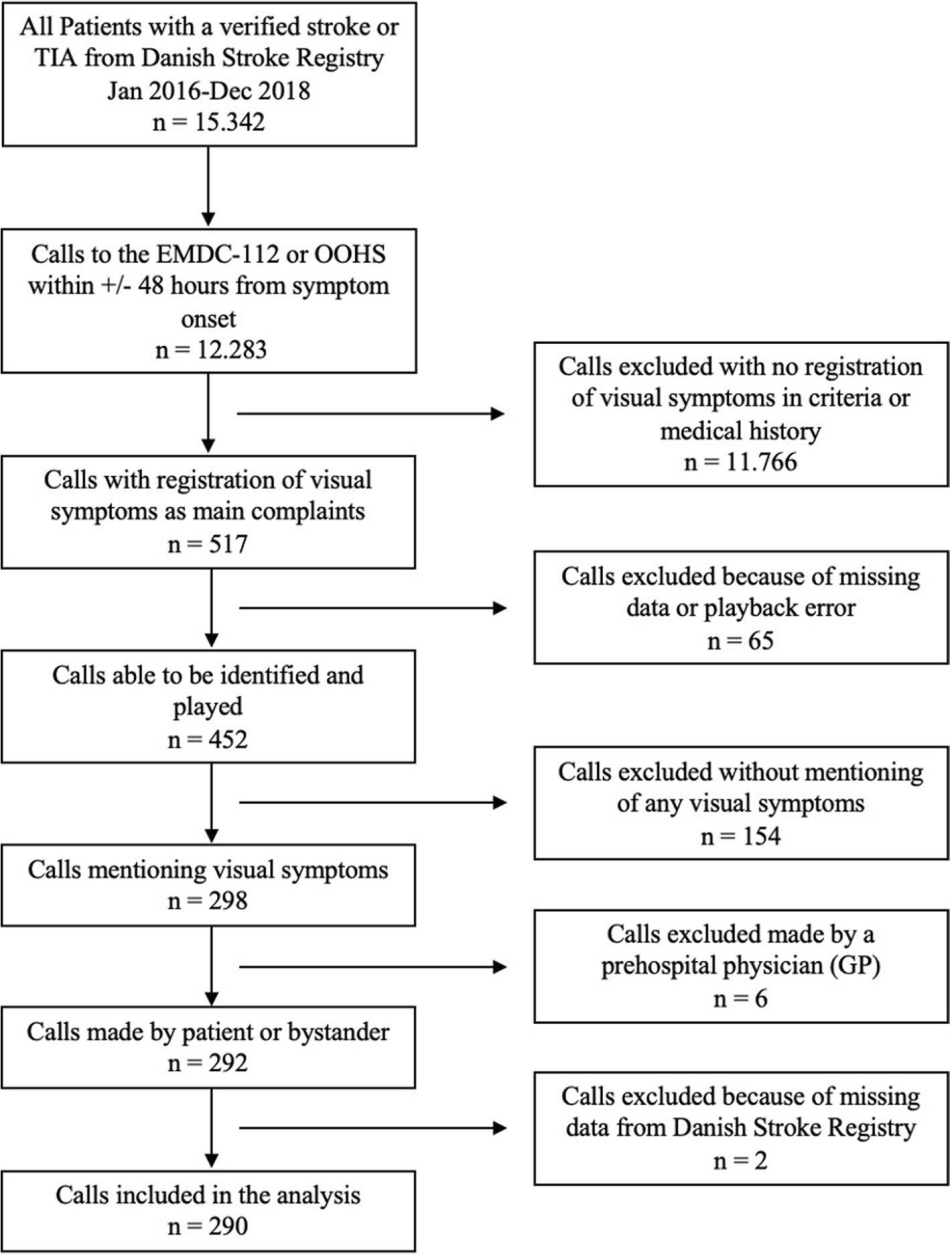
Enrolment of calls from stroke patients with visual symptoms in the Capital Region: a total of 290 calls were included from January 2016 to December 2018

We identified 517 calls to the EMDC-112 or OOHS with a registration of a verified stroke/TIA diagnosis and visual symptoms as main complaints. Of the 517 calls, 290 calls were included in the analysis (Fig. 1).

Of the included patients, 101 (35%) contacted the EMDC-112 and 189 (65%) the OOHS. According to the DANSTROKE registry the contact occurred within 4.5 h from symptom onset in 61% of the patients, and in 23% after 4.5 h from symptom onset. The mean age in both groups was 67.4 (SD = 14.2), and 163 (56%) were male (see Table 1 for demographics between groups).

**Table 1:** Baseline characteristics for the stroke patients

	**Time from symptom onset to hospitalization (h)**			
**Characteristics**	** < 4.5 (*n* = 178)**	** > 4.5(*n* = 112)**	**Total (*n* = 290)**	***p*-value**
**Age, years ± SD**	66.3 ± 14.6	69.1 ± 13.5	67.4 ± 14.2	0.11
< 60 (n = 76)	48.5 ± 9.3 (n = 53)	49.4 ± 10.5 (n = 23)	48.7 ± 9.6	0.69
60–80 (n = 168)	70.5 ± 5.6 (n = 98)	70.6 ± 5.5 (n = 70)	70.6 ± 5.6	0.91
> 80 (n = 46)	86.2 ± 5.0 (n = 27)	87.2 ± 5.1 (n = 19)	86.6 ± 5.0	0.53
**Gender, n (%)**				
Female	72 (40.4)	55 (49.1)	127	-
Male	106 (59.6)	57 (50.9)	163	0.145
**Previous stroke, n (%)**				
No	149 (83.7)	87 (77.7)	236	-
Yes	29 (16.3)	25 (22.3)	54	0.199
**Stroke subtype, n (%)**				
Ischemic	81 (45.5)	70 (62.5)	151	0.005
Hemorrhagic	13 (7.3)	4 (3.6)	17	0.188
TIA	83 (46.6)	37 (33.0)	120	0.022
Missing^a^	1 (0.6)	1 (0.9)	2	-
**Thrombolysis, n (%)**				
No	142 (79.8)	112 (100.0)	254	-
Yes	36 (20.2)	0 (0.0)	36	< 0.0001

^a^Stroke subtype missing from DANSTROKE

Table 1 demographics for patients arriving at the hospital within and after 4.5 h from symptom onset.

Approximately 50% of the calls were done by the patient. The medical dispatchers from the EMDC-112 or OOHS assigned a stroke relevant criterion in 135 (45%) of the calls. A total of 155 patients (53%) were referred to the hospital by ambulance, of which 116 (75%) were a high-priority ambulance (Ambulance A). The remaining 135 patients (47%) were referred to other types of admissions, hereof 38 by urgent ambulance within 25 min (Ambulance B) and one by non-urgent patient-transport ambulance (Ambulance C). Five patients were asked to call back in case of worsening or remittance of symptoms.

Several factors were associated with hospitalization within 4.5 h from symptom onset (Table 2):

**Table 2:** Baseline characteristics for the recorded calls

	**Time from symptom onset to hospitalization (h)**			
**Characteristics**	** < 4.5 (*n* = 178)**	** > 4.5 (*n* = 112)**	**Total (*n* = 290)**	***p*-value**
**Calls to, n (%)**				
OOHS	108 (60.7)	81 (72.3)	189	-
EMDC-112	70 (39.3)	31 (27.7)	101	0.043
**Caller, n (%)**				
Patient	82 (46.1)	62 (55.3)	144	0.187
Relative/friend/stranger	85 (47.7)	45 (40.2)	130	0.185
Healthcare professional	10 (5.6)	4 (3.6)	14	0.577
Other	1 (0.6)	1 (0.9)	2	1.000
**Length of call, n (%)**				
< 5 min	109 (61.2)	62 (55.3)	171	0.145
5–10 min	49 (27.6)	30 (26.8)	79	0.687
> 10 min	20 (11.2)	20 (17.9)	40	0.162
**Time: symptom onset to call, h**				
-4.5 to -48	5 (2.8)	6 (5.3)	11	0.349
0 to -4.5	30 (16.8)	5 (4.5)	35	0.001
0 to 4.5	142 (79.8)	35 (31.3)	177	< 0.0001
4.5 to 48	1 (0.6)	66 (58.9)	67	< 0.0001
**Dispatcher referral to, n (%)**				
Ambulance A	94 (52.8)	22 (19.6)	116	< 0.0001
Ambulance B	18 (10.1)	20 (17.9)	38	0.076
Ambulance C	1 (0.6)	0 (0.0)	1	1.0
Emergency dept	46 (25.8)	42 (37.5)	88	0.051
Planned admission	3 (1.7)	14 (12.3)	17	0.0004
Ophthalmologist	0 (0.0)	2 (1.8)	2	0.152
Own GP	10 (5.6)	4 (3.6)	14	0.577
Selfcare	2 (1.2)	3 (2.7)	5	0.382
Missing	4 (2.2)	5 (4.6)	9	-
**Assigned stroke relevant criterion, n (%)**				
No	70 (39.3)	65 (58.0)	135	-
Yes	94 (52.8)	37 (33.0)	131	0.001
Missing	14 (7.9)	10 (9.0)	24	-

Firstly, contacting the EMDC-112 compared to contacting the OOHS (*p* = 0.043), secondly, making the call for help within 4.5 h from symptom onset (*p* < 0.0001), thirdly, when the patients were referred to the hospital by a high-priority ambulance (*p* < 0.0001), and lastly, when the medical dispatchers assigned the patients a stroke relevant criterion compared to no stroke relevant criterion (*p* = 0.001). Thrombolysis was only given to patients arriving at the hospital within 4.5 h from symptom onset (*p* < 0.0001). Delay factors were if the call was done after 4.5 h from symptom onset (*p* < 0.0001), and if the patients were referred to planned admission with scheduled admission time (*p* < 0.0004).

Table 2 call characteristics for patients arriving at the hospital within and after 4.5 h from symptom onset.

**Fig. 2:**
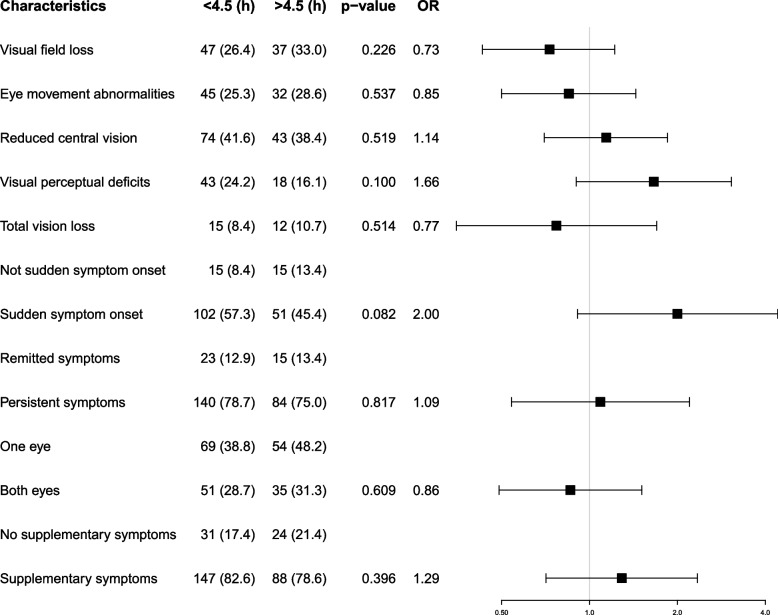
Incidence and characteristics of visual symptoms. Data is summarized as absolute numbers and percentages with P-value, odds ratio (OR) and 95% confidence interval (CI) plots. For the five overall visual symptoms (the top five lines), the OR and 95% CI were calculated using the patients not experiencing the specific visual symptom as reference. For the visual characteristics (last eight), the OR and 95% CI were calculated using the opposite characteristic as reference, e.g. *No supplementary symptoms* as reference to *Supplementary symptoms*, thus generating one overall p-value, OR and 95% CI

Characteristics of the visual symptoms identified from the calls were compared between the patients arriving at the hospital within and after 4.5 h from symptom onset (Fig. 2).

The most frequently reported visual symptom was that of reduced central vision (40%). Also, acknowledgement of visual field loss (29%), eye movement abnormalities (27%), and visual perceptual deficits (21%) were frequently reported. Total vision loss in one or both eyes was less common (10%). Some patients reported more than one visual symptom during the call. The visual symptoms were most often reported with acute onset (53%), located to one eye (42%), and were persistent (77%). A total of 55 patients only experienced visual symptoms, whereas the remaining (81%) reported one or more supplementary symptoms (Supplementary material—Figure S1). Some of the visual symptoms and characteristics had an OR higher than 1, but none of the results were significant associated with early hospitalization.

A univariate logistic analysis was performed, and positive correlation was found between correct visitation and contact within 4.5 h from symptom onset to either EMDC-112 or OOHS with an OR of 2.54 [1.29, 5.02] (Table 3).

**Table 3:** Logistic regression analyses between correct visitation and multiple variables

**Univariate logistic analysis**			
**Variables**	**SE**	***p*-value**	**OR [95% CI]**
Intercept	0.318	< 0.0001	0.21 [0.01, 0.35]
Contact within 4.5 h from symptom onset	0.348	0.007	2.54 [1.29, 5.02]
**Multivariate logistic analysis**			
**Variables**	**SE**	***p*-value**	**OR [95% CI]**
Intercept	0.969	0.002	0.05 [0.03, 0.78]
Contact within 4.5 h from symptom onset	0.384	0.025	2.37 [1.11, 5.03]
Call to EMDC-112	0.291	< 0.0001	3.18 [1.80, 5.62]
Age	0.010	0.825	1.00 [0.98, 1.02]
Gender	0.289	0.570	1.18 [0.67, 2.08]
Previous stroke	0.370	0.212	1.59 [0.77, 3.28]
Stroke suspected by caller	0.309	0.363	0.76 [0.41, 1.38]
Supplementary symptoms	0.364	0.257	0.66 [0.32, 1.35]
Questions about typical stroke symptoms	0.382	< 0.0001	6.36 [3.01, 13.43]

Table 3 univariate- and multivariate logistic regression analyses. A standard error (SE), p-value, odds ratio (OR) and 95% confidence interval (CI) are given.

In a supplementary multivariate logistic analysis, the following confounders were applied: Call to the EMDC-112, age, gender, previous stroke, stroke suspicion by the caller, supplementary symptoms, and supplementary questions from the medical dispatchers on typical stroke symptoms. A positive correlation was found between correct visitation and contact within 4.5 h from symptom onset with an adjusted OR of 2.37 [1.11, 5.03]. A positive correlation was also seen between correct visitation and contact to the EMDC-112 with an adjusted OR of 3.18 [1.8, 5.62], and when the medical dispatcher asked questions on typical stroke symptoms with an adjusted OR of 6.36 [3.01, 13.43]. None of the other variables showed significant association with correct visitation.

### Inter-rater reliability and optimization of telephone stroke data form

Three raters with health professional background evaluated 25 calls, blinded for each other’s evaluation. To determine the inter-rater reliability an initial Kappa Fleiss score was calculated [24] and the Telephone Stroke Data Form was modified to improve inter-rater agreement. This procedure was done twice on the same 25 calls, resulting in overall good agreement [24].

## Discussion

In this study we found that only 30% of the stroke patients who experienced visual symptoms received correct visitation with an assigned stroke relevant criterion by the dispatcher, and referral to the hospital by a high-priority ambulance. Correct visitation was significantly associated with early contact after symptom onset, first contact to the EMDC-112, and when the medical dispatchers asked additional questions on typical stroke symptoms. Overall, results confirmed our hypothesis that recognizing visual symptoms as a sign of acute stroke was challenging for both patients and medical dispatchers. Both parties did not know how to respond appropriately to stroke with mainly visual symptoms, indicating little knowledge of the national stroke guidelines. Further, none of the reported visual symptoms showed specific correlation with stroke recognition and fast hospitalization. We did not find any significant differences in the types of visual symptoms experienced by the patients who arrived at the hospital within or after 4.5 h from symptom onset. Also, the reported visual symptoms were wide-ranging and often nonspecific. We did not find that the occurrence of supplementary atypical symptoms concomitant to the visual symptoms improved identification of stroke by the medical dispatchers, and the appearance of multiple stroke symptoms did not associate with correct visitation.

This study is the first to investigate the association between visual symptoms reported in calls to the EMDC-112 or OOHS, and prehospital diagnosis of acute stroke. Several limitations may apply to the study. There is a risk of missing patients presenting with visual symptoms, if not registered with visual symptoms. To reduce the risk of losing patients, patients with visual symptoms were identified by the use of both registration and a free-text search of dispatch notes for words referring to vision. When analyzing the reported visual symptoms, it could be difficult to place the visual symptom into one of the five overall categories. If the patients reported unspecific visual symptoms, the visual symptoms were registered by the raters as reduced central vision in the Telephone Stroke Data Form. This procedure may cause an overestimation of central vision symptoms. All supplementary symptoms reported by the patients were registered. The medical dispatchers inquired about other symptoms, which could have caused the patients to report symptoms not otherwise detected, including some that were not stroke related. Secondly, we only included patients who made contact to the EMDC-112 or OOHS. Patients who contacted their general practitioner or others for help and patients who self-presented at the emergency department were not included. Finally, the data from DANSTROKE included an estimated time of symptom onset. In these data, the estimated time of symptom onset was registered after the call and may thus be subject to recall bias. Such can explain discrepancy in symptom onset and call time with the latter occurring before symptom onset and relate to a registration error.

In our study almost 40% of the stroke patients who experienced visual symptoms did not arrive at the hospital in time to receive revascularization treatment. Main barriers for fast hospitalization were previously identified as patient-dependent and prehospital delay [25, 26]. Patient-dependent delay were associated with reduced knowledge or failed recognition of stroke symptoms [4, 25, 27–29]. We found that 73% of the patients contacted the EMDC-112 or OOHS within 4.5 h from symptom onset; thus, most of the patients had a fast response to onset of visual symptoms, irrespective of a stroke being suspected. However, one in four of the patients in our study did not react to the new symptoms by seeking help. This suggest either little knowledge of visual symptoms as a sign of stroke, or that the visual symptoms went unnoticed by the patients, or they suspected other and less serious causes of the visual disturbances [4]. Other studies reported that stroke patients had difficulty recognizing atypical stroke symptoms including visual symptoms [3, 29–31]. The patients were more likely to recognize the typical stroke symptoms such as speech difficulties, unilateral symptoms, and facial drooping [3, 29–31]. An important factor reported in patient-dependent delay was reduced knowledge on how to act on stroke symptoms [25–29, 32, 33]. This is a key dilemma in acute stroke treatment; though patients recognize the stroke symptoms in time, they do not act appropriately on the symptoms. We found that first contact to the EMDC-112 was associated with correct visitation with rapid hospitalization, and prehospital delay may thus relate to no initial contact to the EMDC-112 [5–8, 25, 33–36]. In a previous Danish study up to 82% of the participants reported that they would contact EMDC-112, if they experienced stroke symptoms [37]. However, we found that only 35% of the patients contacted the EMDC-112.

Prehospital delay may also be caused by poor stroke recognition by medical dispatchers [12, 13, 25, 31]. This is consistent with our findings where only 45% of the patients were recognized and assigned a stroke relevant criterion by the dispatcher. In other studies, the medical dispatchers were more likely to recognize a stroke and initiate fast intervention if the patients experienced one or more of the typical stroke symptoms [10, 11]. Though stroke may be recognized correctly, prehospital delays are caused by incorrect choice in chain-of-referral for stroke patients by the medical dispatchers [25, 26]. Despite early contact after symptom onset to the EMS, we found that a rather large proportion of the patients arrived at the hospital after 4.5 h from symptom onset, which could be associated to the finding that only 40% of the patients were referred to the hospital by a high-priority ambulance. The remaining patients were referred to the emergency department, planned admission or their usual general practitioner for further examination [5–8, 25–27, 34].

## Conclusion

Both patients and medical dispatchers had little knowledge on optimal response to stroke symptoms, which highlight that more information on the correct chain-of-response to stroke is needed.

It is crucial that stroke patients who experience visual symptoms receive correct visitation by first line healthcare professionals to minimize delayed hospitalization and increase the number of patients eligible for revascularization treatment. Continued education on visual stroke symptoms and appropriate patient- and dispatcher behavior towards timely treatment, is essential.

### Supplementary Information

**Additional file 1.**

## Data Availability

De-identified datasets used during the current study are available from the corresponding author upon reasonable request.
