# Supplementary material for: Recognition of visual symptoms in stroke: a challenge to patients, bystanders, and Emergency Medical Services
Source: BMC Emerg Med. 2023 Aug 25;23:96. doi: 10.1186/s12873-023-00870-2 (PMC10463357; doi:10.1186/s12873-023-00870-2)
Supplement: Supplementary file 1 — Additional file 1. [file 12873_2023_870_MOESM1_ESM.docx]

Figure S1. Distribution of supplementary non-visual symptoms experienced by the patients.
